# Single-lead ECG based autonomic nervous system assessment for meditation monitoring

**DOI:** 10.1038/s41598-022-27121-x

**Published:** 2022-12-29

**Authors:** Chanki Park, Inchan Youn, Sungmin Han

**Affiliations:** 1 Future and Basic Technology Research Division, ICT Creative Research Laboratory, Electronics and Telecommunications Research Institute, CybreBrain Research Section, Daejeon, 34129 Republic of Korea; 2 Bionics Research Center, Biomedical Research Division, Korea Institute of Science and Technology, Seoul, 02792 Republic of Korea; 3 Division of Bio‑Medical Science and Technology, Korea Institute of Science and Technology School, Seoul, 02792 Republic of Korea; 4 KHU-KIST Department of Converging Science and Technology, Kyung Hee University, Seoul, Seongbuk-gu 02447 Republic of Korea

**Keywords:** Biomarkers, Health care, Biomedical engineering

## Abstract

We propose a single-lead ECG-based heart rate variability (HRV) analysis algorithm to quantify autonomic nervous system activity during meditation. Respiratory sinus arrhythmia (RSA) induced by breathing is a dominant component of HRV, but its frequency depends on an individual’s breathing speed. To address this RSA issue, we designed a novel HRV tachogram decomposition algorithm and new HRV indices. The proposed method was validated by using a simulation, and applied to our experimental (mindfulness meditation) data and the WESAD open-source data. During meditation, our proposed HRV indices related to vagal and sympathetic tones were significantly increased (p < 0.000005) and decreased (p < 0.000005), respectively. These results were consistent with self-reports and experimental protocols, and identified parasympathetic activation and sympathetic inhibition during meditation. In conclusion, the proposed method successfully assessed autonomic nervous system activity during meditation when respiration influences disrupted classical HRV. The proposed method can be considered a reliable approach to quantify autonomic nervous system activity.

## Introduction

Due to the increase in the number of mental disorders, various mental healthcare therapies (e.g., psychotherapy, digital therapeutics, and meditation) have been widely used to improve emotional wellness^1^. Over the past few decades, mindfulness meditation has drawn much attention because of its benefits, such as emotion regulation, increased awareness, and improved cognitive performance^2–4^, and it has been clinically used to reduce chronic pain, sleep disturbance, anxiety, distress, and depression^5–7^. Many neuroscientific studies have investigated changes in the brain during meditation^2^, and the activation of several brain regions, such as the frontopolar cortex, sensory cortex, insula, hippocampus, anterior cingulate cortex, mid-cingulate cortex, and orbitofrontal cortex, has been observed^8^. These brain regions are related to meta-awareness, body awareness, memory processes, and emotion regulation. In particular, the insular and mid-cingulate cortices play central roles in the central autonomic network, which controls the activity of parasympathetic and sympathetic nerves^9^. Through this neurological pathway, it seems that body relaxation is induced during meditation.

**Figure 1:**
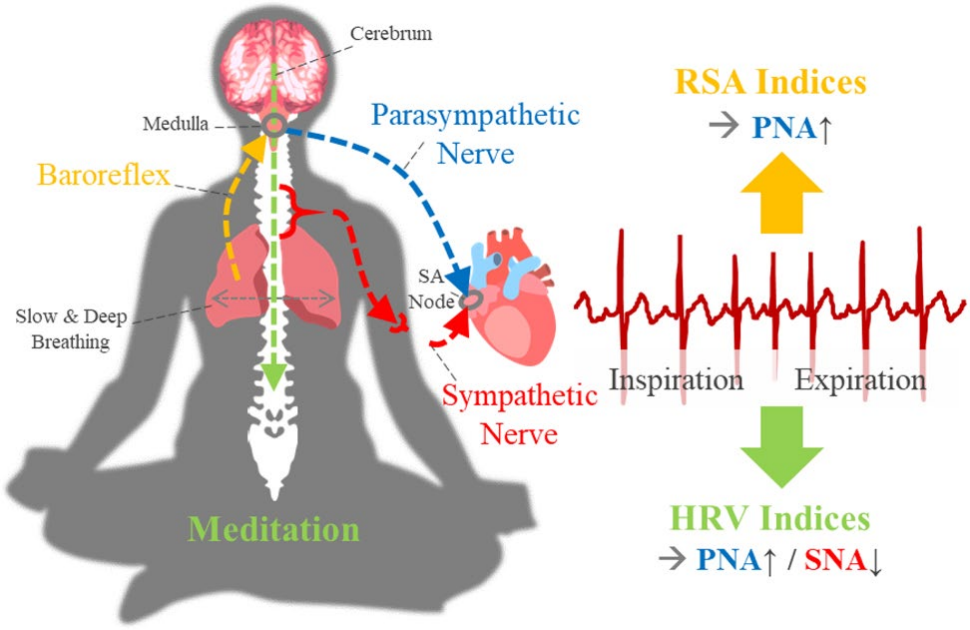
Meditation and single-lead ECG.

As a technology-based therapy, biofeedback via a wearable device has been developed^10^. Biofeedback devices help users carry out a therapeutic protocol (e.g., meditation) by means of their physiological signals as feedback. Electroencephalogram-based neurofeedback training is a representative biofeedback therapy that is used to treat various mental disorders, such as insomnia, anxiety, depression, and addiction^11^. On the other hand, electroencephalogram-based digital meditation was proposed to enhance attention and working memory^12,13^. Although neurofeedback treatment shows various clinical effects, an electroencephalogram sensor is inconvenient. Recently, a new modality called skin sympathetic nerve activity has been developed to noninvasively observe the activity of sympathetic nerves^14,15^. It can be simply recorded from the high frequency component (500–1000 Hz) of an analog electrocardiogram (ECG), but it is very vulnerable to motion artifacts due to its weak amplitude^16,17^. Because it is easy to measure ECG signals with a high signal-to-noise ratio in daily life, heart rate variability (HRV)-based biofeedback training is drawing attention^18,19^. In this study, we propose a single-lead ECG-based HRV analysis algorithm to investigate the changes in vagal and sympathetic tones during meditation (see Fig. 1).

ECG based cardiopulmonary coupling analysis and HRV analysis have been used to assess psychiatric conditions. Cardiopulmonary coupling analysis measures the synchronization between the heartbeat interval and respiration by using the spectral coherence, cross entropy and phase locking value (PLV)^20–22^. This synchronization phenomenon is called respiratory sinus arrhythmia (RSA), which is the modulation of efferent parasympathetic nerves caused by baroreflexes (arterial baroreflex, lung stretch reflex, and Bainbridge reflex) during the inspiration phase^23–26^. RSA is affected by the tidal volume and respiratory rate (RR)^27–29^, and its quantity reflects sleep quality and apnea^20,22^. To capture other neurophysiological changes, HRV is widely used, and its frequency analysis reflects the balance between vagal and sympathetic tones. The former shows a fast response, and the latter dominates the low-frequency band of HRV and seems to be independent of the baroreflex^30^.

Meditation (or relaxation) training leads to parasympathetic activation and sympathetic inhibition, and it is supported by several modalities, such as muscle sympathetic nerve activity^31^, functional MRI^2^, and electroencephalogram^32^. Some HRV studies on meditation also reported an increase in high-frequency power^32–35^, which indicates vagal tone, but other studies showed the opposite results^36–40^. These contradictory results seem to be due to the influence of RSA. When the RR < 0.15 Hz, the RSA component moves from the high- to low-frequency region, and then the low- and high-frequency powers can be overestimated and underestimated, respectively^28^. Hence, the fluctuation of an individual’s RR led to significant variation in HRV analysis^41^, and there was no report on the identification of cardiac sympathetic inhibition during meditation.

**Figure 2:**
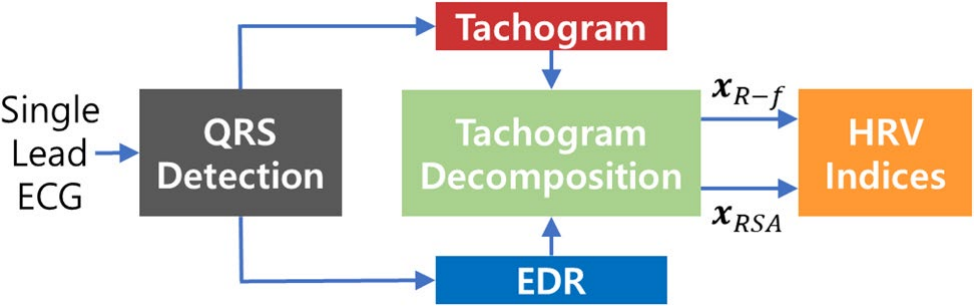
Overall structure of the proposed method.

To separate the RSA effect and other autonomic nervous system activities in HRV analysis, various decomposition algorithms have been utilized, such as independent component analysis^42^, adaptive noise cancellers^43^, autoregressive moving average with exogenous input model (ARMAx)^44^, and orthogonal subspace projection (OSP)^45,46^. The adaptive noise canceller, ARMAx, and OSP share the same algorithmic structure, with OSP exhibiting the best performance^45^. Even though these algorithms significantly enhance the reliability of HRV, they assume a linear relationship between the RSA component and a respiration signal, while in reality the relationship is nonlinear^47^. As an alternative, a notch filter with a Gaussian bell shape (Gauss) was employed, but it assumed that the instantaneous RR is constant^48^. In this study, to overcome the nonlinearity and nonstationarity of RSA, we proposed a novel HRV tachogram decomposition algorithm, and new HRV indices to quantify vagal and sympathetic tones during meditation (see Fig. 2).

The proposed method is described in the “Materials and methods” section. We compared the performance of decomposition algorithms using simulation, and observed the changes in the vagal and sympathetic tones during meditation through real ECG data in “Results” section. In “Discussion” section, the results are discussed.

## Materials and methods

Since HRV reflects the balance of the autonomic nervous system, HRV analysis is frequently used to assess stress and psychiatric conditions. The first step of HRV analysis is QRS detection on the ECG. In this study, we bandpass filtered an ECG with 0.5–50 Hz by a 3rd-order Butterworth, and we employed the Pan-Tompkins algorithm to detect R-peaks of the ECG^49^. The interval between successive R-peaks (RRI) is accumulated over a predefined time window. In this study, we adopted a 5 min time window for short-term HRV analysis. To detect ectopic beats and incorrect peaks, we calculated the *k*-th derivative of the instantaneous heart rate (HR)^50^ as follows:

1 $${HR\mathrm{^{\prime}}}_{k}=2\left|\frac{{R}_{k-1}-2{R}_{k}+{R}_{k+1}}{({R}_{k-1}-{R}_{k})({R}_{k-1}-{R}_{k+1})({R}_{k}-{R}_{k+1})}\right|$$

where *R*_*k*_ is the *k*-th R-peak. When an *HR’*_*k*_ was greater than a predefined threshold, *R*_*k*_ was excluded from the HRV analysis, and the corrected R-peaks were double-checked by human experts. Because the RRI is sampled at irregular time points (heartbeats), the RRI must be converted into an evenly sampled time series for frequency analysis. A uniformly sampled signal of the RRI, called a tachogram, was reconstructed using cubic spline interpolation, and we resampled the tachogram to 4 Hz, as in previous studies^44–46^. For precise HRV analysis, it is necessary to distinguish the cardiac vagal tone modulated by respiration and other autonomic nervous system activities. Hence, we consider the original tachogram ***x***_*RAW*_ as follows:

2 $$x_{RAW} = {\mathbf{x}}_{RSA} + {\mathbf{x}}_{R - f}$$

where ***x***_*RSA*_ and ***x***_*R-f*_ are the RSA and RSA-free tachograms, respectively. ***x***_*R-f*_ is obtained by removing ***x***_*RSA*_ from ***x***_*RAW*_. The tachogram decomposition algorithm will be described in the next subsection. In this study, since we focus on the tachogram decomposition algorithm, we adopt only frequency domain HRV indices.

### ECG-derived respiration

To measure the respiration signal, various sensors have been utilized, such as a pressure transducer and a thermistor. These sensors provide accurate information but are both expensive and inconvenient to wear. As an alternative, several algorithms to estimate the respiration signal from biomedical signals, such as photoplethysmogram and ECG, have been proposed^51–54^. In this study, we employed an ECG-derived respiration (EDR) algorithm that captures the change in electrical impedance and the cardiac vector according to chest expansion. Here, we briefly review 4 frequently used EDR algorithms: EDR_A_ (R-wave amplitude), EDR_QR_ (Q-R slope), EDR_RS_ (R-S slope) and EDR_SR_ (slope range). Each EDR algorithm uses a respiration-related sample r(i) at the i-th heartbeat as follows:

- EDR_A_: r_A_(*i*) = R-peak amplitude
- EDR_QR_: r_QR_(*i*) = steepest ascent slope on Q-R wave
- EDR_RS_: r_RS_(*i*) = steepest descent slope on R-S wave
- EDR_SR_: r_SR_(*i*) = r_QR_(*i*) − r_RS_(*i*)

By interpolating the respiration-related samples, the EDR signal is generated. The overall procedure is explained in detail in^51^.

### Tachogram decomposition

#### Previous algorithms

To decompose raw tachogram ***x***_*RAW*_ into ***x***_*RSA*_ and ***x***_*R-f*_, various algorithms have been proposed, such as Gauss, ARMAx and OSP. Both the ARMAx and OSP algorithms assume ***x***_*RSA*_ as follows:

3 $$\overline{\user2{x}}_{RSA} = RESP\left( {RESP^{T} \cdot RESP} \right)^{{ - {1}}} RESP \cdot {\mathbf{x}}_{RAW}$$

where *RESP* is a respiratory basis matrix. The trajectory matrix^46^ or wavelet coefficients^45^ of EDR have been utilized as the *RESP* matrix. In this study, the trajectory matrix was employed for the *RESP*. We assigned 3 s to the lag size of the trajectory matrix of the ARMAx, while the lag size of OSP was set to the minimum value between the minimum description length and the Akaike information criterion, with a maximum lag of 10 s. The RSA-free tachogram is estimated as follows:

4 $${\overline{{\varvec{x}}} }_{R-f}={{\varvec{x}}}_{RAW}-{\overline{{\varvec{x}}} }_{RSA}$$

The ARMAx and the OSP are described in^44–46^ in detail. As another tachogram decomposition algorithm, a notch filter with Gaussian bell shape (Gauss) was proposed. Its magnitude-squared frequency response is:

5 $${\left|H\left({e}^{j\omega },\theta \right)\right|}^{2}=\alpha \cdot \left(1-{e}^{-\beta \cdot {(\omega -\theta /fs)}^{2}}\right)$$

where *θ* represents the notch frequency and corresponds to the average RR. *α* and *β* are fitting parameters and it can be computed from the spectrum of ***x***_*RAW*_ by using a least square method. The filtered signal will be ${\overline{{\varvec{x}}} }_{R-f}$, and ${\overline{{\varvec{x}}} }_{RSA}$ is acquired by subtracting ${\overline{{\varvec{x}}} }_{R-f}$ from ***x***_*RAW*_. The overall procedure of Gauss is described in^48^.

#### Proposed algorithm

**Figure 3:**
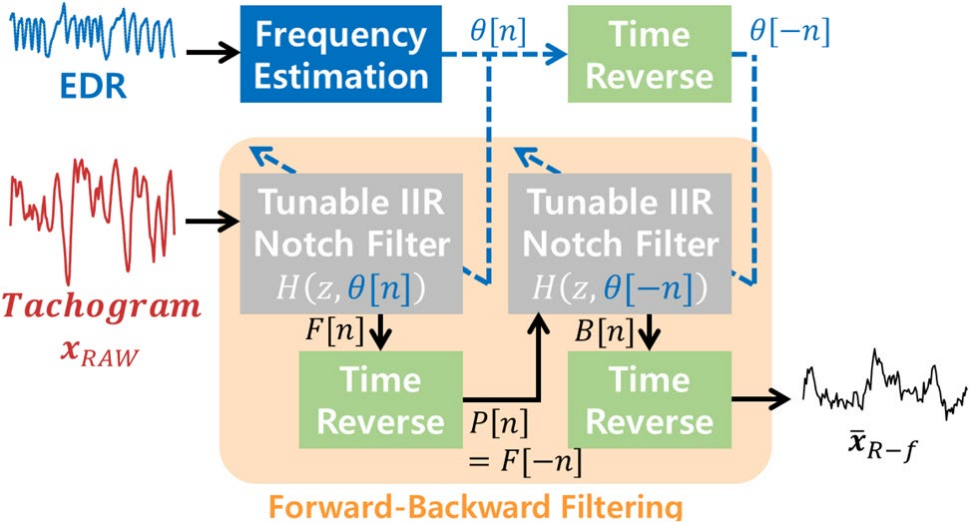
Tachogram decomposition process of ZLE. The ZLE is composed of (**a**) instantaneous RR *θ*[*n*] estimation and (**b**) time-variant forward–backward IIR notch filtering.


Based on the synchronization between the frequency of ***x***_*RSA*_ and the instantaneous RR, we designed a novel tachogram decomposition algorithm. Because it has a zero-phase response, we call it the zero-phase line enhancer (ZLE). The ZLE can be interpreted as an adaptive filter without phase distortion^55^. As shown in Fig. 3, the ZLE has two parts: (a) instantaneous RR estimation and (b) time-variant forward–backward IIR notch filtering. To estimate the instantaneous RR, we employed a smoothed pseudo Wigner–Ville distribution of the EDR signal^56,57^ as follows:

6 $$\omega \left[k,n\right]=\sum_{m=-N}^{N}g\left[n\right]v\left[m\right]EDR\left[n+\frac{m}{2}\right]{EDR}^{*}\left[n-\frac{m}{2}\right]{e}^{\frac{-j2\pi km}{N}}$$

where N represents the length of the EDR (N = 5 min∙60∙4 Hz). *g*[] and *v*[] are the time and frequency windows, respectively; a Kaiser window (γ = 20) was employed for *g*[] and *v*[]. From the time–frequency representation *ω*[*k*, *n*], we estimated the instantaneous RR *θ*[*n*] by using a recursive algorithm to minimize the problem of outliers. The maximum point among frequency bins adjacent to *θ*[*n*-1] was assigned to *θ*[*n*], as described in Algorithm 1.

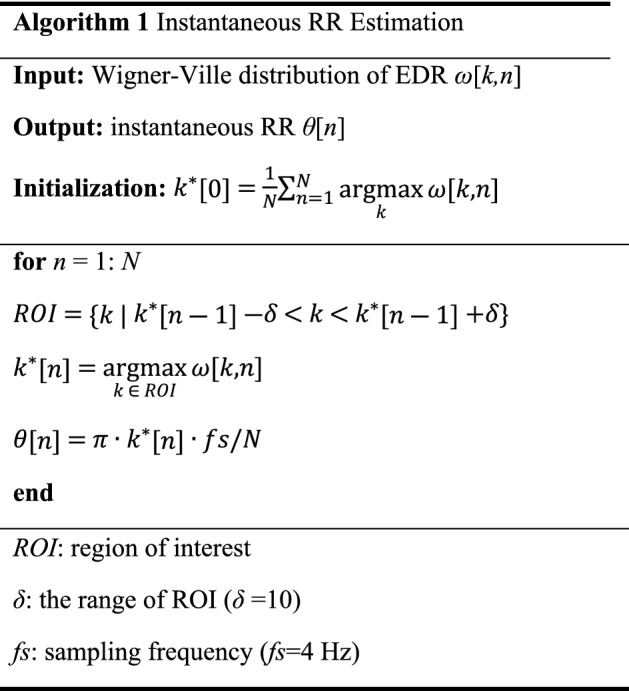


The frequency component of ***x***_*RAW*_ corresponding to the instantaneous RR *θ*[*n*] will be ***x***_*RSA*_, and the ZLE removes ***x***_*RSA*_ by means of a tunable IIR notch filter^58^ as follows:

7 $$H\left(z,\theta [n]\right)=\frac{1+r}{2}\frac{1-2\mathit{cos}\left(\theta \left[n\right]/fs\right){z}^{-1}+{z}^{-2}}{1-(1+r)\mathit{cos}\left(\theta [n]/fs\right){z}^{-1}+r{z}^{-2}}$$

where *r* is the pole-zero contraction factor, which determines the stop band. When *r* is closer to 1, the frequency response of the notch filter becomes narrower. We set *r* to 0.93, and then the stop band of *H*(*z*, *θ*[*n*]) is 0.047 Hz. As shown in Fig. 3, the time-variant forward–backward IIR notch filtering performs four sequential steps: 1—forward IIR notch filtering, 2—time reversal, 3—backward IIR notch filtering, and 4—time reversal. The zero-phase response can be explained in the z-domain as follows:

8 $$F(z)=H\left(z,\theta \left[n\right]\right)\cdot {X}_{RAW}(z)$$

9 $$P\left(z\right)={H}^{*}\left(z,\theta \left[-n\right]\right)\cdot {{X}^{*}}_{RAW}(z)$$

10 $$B\left(z\right)=H\left(z,\theta \left[-n\right]\right)\cdot {H}^{*}\left(z,\theta \left[-n\right]\right)\cdot {{X}^{*}}_{RAW}(z)$$

$$={\left|H\left(z,\theta \left[-n\right]\right)\right|}^{2}\cdot {{X}^{*}}_{RAW}(z)$$

11 $${\overline{X} }_{R-f}(z)={\left|H\left(z,\theta \left[n\right]\right)\right|}^{2}\cdot {X}_{RAW}(z)$$

where F(z) and B(z) mean forward- and backward-filtered signals, respectively. P(z) represents the time-reversed F(z). The filter output is the estimated RSA-free tachogram ${\overline{{\varvec{x}}} }_{R-f}$, and the RSA tachogram is estimated as follows:

12 $${\overline{x} }_{RSA}={x}_{RAW}-{\overline{x} }_{R-f}$$

### RSA indices

RSA has been utilized as a measure of cardiac vagal function. RSA can be quantified by cardiopulmonary coupling analysis. In this study, we utilized the PLV^22,39,59^. The PLV is broadly used to measure a phase interaction between two signals and is defined as follows:

13 $$PLV=\left|\frac{1}{ N}\sum_{n=1}^{N}{e}^{j[{\varphi }_{1}\left(n\right)-{\varphi }_{2}(n)]}\right|$$

where *φ*_1_(n) and *φ*_2_(n) represent the phases of the respiration signal (or EDR) and tachogram ***x***_***RAW***_, respectively. The phases are calculated by a Hilbert transform. As another measure for RSA, the extent of RSA (RSA_E_)^27,39,60^ was calculated by the median value of RRI variation (longest RRI—shortest RRI) within each breathing cycle as follows:

14 $${RSA}_{E}=med\left({Var}_{RRI}\right)$$

15 $${Var}_{RRI}=\left\{\mathrm{max}\left({RRI}^{k}\right)-\mathrm{min}\left({RRI}^{k}\right)\right|k=\mathrm{1,2},\dots ,K\}$$

where *RRI*^k^ represents the subset of *RRI*s within the *k*-th breathing cycle. max and min are maximum and minimum functions, respectively. *K* indicates the number of breaths taken in the time window (5 min). Because RSA_E_ is based on the RRI, the unit of RSA_E_ is ms.

### HRV indices

In HRV analysis, the power spectrum of ***x***_*RAW*_ is segmented into low-frequency (0.04–0.15 Hz) and high-frequency (0.15–0.4 Hz) bands, and each band power is called LF (low frequency) and HF (high frequency)^61^. HF is regarded as an index for vagal tone, and LF reflects both sympathetic and vagal tones. To quantify sympathetic tone, LF/HF is generally used. Since these HRV indices were insufficient to cover the aforementioned RSA issue, it is necessary to simultaneously use both power spectra of ${\overline{{\varvec{x}}} }_{R-f}$ and ${\overline{{\varvec{x}}} }_{RSA}$; Subscripts *R-f* and *RSA* were used to distinguish each band power (${\overline{{\varvec{x}}} }_{R-f}$: LF_*R-f*_ and HF_*R-f*_, ${\overline{{\varvec{x}}} }_{RSA}$: LF_*RSA*_ and HF_*RSA*_). To assess sympathovagal balance, Varon et al. designed an HRV index SB_U_ based on the separated band powers^46^ as follows:

16 $${\text{SB}}_{{\text{U}}} = {\text{LF}}_{R - f} /\left( {{\text{LF}}_{RSA} + {\text{HF}}_{RSA} } \right)$$

Since ***x***_*RSA*_ and HF_*R-f*_ purely indicate vagal tone but LF_*R-f*_ is related to both vagal and sympathetic tones, we proposed the new HRV indices related to vagal (Eq. 17) and sympathetic (Eq. 18) tones as follows:

17 $${\text{IDX}}_{{{\text{PNA}}}} = {\text{HF}}_{R - f} + {\text{LF}}_{RSA} + {\text{HF}}_{RSA}$$

18 $${\text{IDX}}_{{{\text{SNA}}}} = {\text{LF}}_{R - f} /\left( {{\text{HF}}_{R - f} + {\text{LF}}_{RSA} + {\text{HF}}_{RSA} } \right)$$

We employed Welch’s method to compute the power spectra.

### Simulation

To compare the tachogram decomposition algorithms, we designed a simulated tachogram ${\widetilde{{\varvec{x}}}}_{RAW}$. First, to cover various situations, we considered three respiration types as follows:

19 $$\widetilde{EDR}=\mathrm{cos}(2\pi \psi (n))$$

20 $$RR=\dot{\psi }\left(n\right)=\left\{\begin{array}{cc}\textcircled{1}& constant\\ \textcircled{2}& a\cdot n+b\\ \textcircled{3}& a\cdot \mathrm{cos}\left(2\pi \omega n\right)+b\end{array}\right.$$

where $\dot{\psi }\left(n\right)$, the derivative of $\psi (n)$, means the simulated RR. ①, ②, and ③ represent the frequencies of a sine wave (0.15 Hz), linear chirp (0.04–0.4 Hz), and sinusoidal frequency modulated signal (0.04–0.4 Hz). Since not only the frequency but also the amplitude of the tachogram is modulated by RR^27–29^, we devised an amplitude modulation function for the simulated RSA component ${\widetilde{{\varvec{x}}}}_{RSA}$. Specifically, because the amplitude of the tachogram with respect to RR in^29^ was skewed to the right, we utilized the gamma distribution function as the amplitude modulation function *m*() as follows:

21 $${\widetilde{x}}_{RSA}(n)=m(\dot{\psi }(n))\cdot \mathrm{cos}(2\pi \psi (n))$$

22 $$m\left(f\right)=Gamma\left(\alpha ,\beta \right)={\beta }^{\alpha }{(f)}^{\alpha -1}{e}^{-f\cdot \beta }/\Gamma (\alpha )$$

where Г(*α*) is the gamma function. *α* and *β* are shape and rate parameters (*α* = 2, *β* = 15). Note that when $\dot{\psi }\left(n\right)$ is constant the amplitude modulation function *m*() becomes constant. On the other hand, as the simulated RSA-free tachogram $\widetilde{{\varvec{x}}}$_*R-f*_, we employed white Gaussian noise as follows:

23 $${\widetilde{x}}_{R-f}=wGn\left({\sigma }^{2}\right)$$

where ${\sigma }^{2}$ is the variance of the white Gaussian noise. We adjusted ${\sigma }^{2}$ so that the power ratio of $\widetilde{{\varvec{x}}}$_*RSA*_ and $\widetilde{{\varvec{x}}}$_*R-f*_ was equal (0 dB), and then the simulated tachogram $\widetilde{{\varvec{x}}}$_*RAW*_ was given by their summation.

24 $${\widetilde{{\varvec{x}}}}_{RAW}={\widetilde{{\varvec{x}}}}_{RSA}+{\widetilde{{\varvec{x}}}}_{R-f}$$

For rigorous comparison, we performed Monte Carlo simulation with 1000 independent simulations.

### Database

To investigate the changes in HRV indices according to the tachogram decomposition algorithms during meditation, we utilized two real ECG databases: mindfulness meditation data and WESAD data.

#### Mindfulness meditation data

We collected a single-lead ECG and a respiration signal from 8 male and 8 female subjects (age = 28.5 ± 4.2 years) during mindfulness meditation. All subjects were novice meditators who had engaged in meditation within 1–2 times. All subjects rested for 10 min and then performed mindfulness meditation for 40 min under the guidance of a meditation teacher. During mindfulness meditation, subjects were guided to maintain awareness of their breathing and sensations without judgment. Breathing speed was not restricted. All subjects closed their eyes and remained in a sitting position to minimize motion artifacts in the ECG. They completed a visual analog scale (VAS) for stress, tension, and concentration before and after mindfulness meditation. Each ECG was measured by a wearable ECG patch (EP200, Life Science Technology^®^, Seoul, Korea), and the sampling rate was 250 Hz. The reference respiration signal was recorded by a respiratory transducer (RSPEC-R, Biopac^®^, California, USA) with a sampling rate of 250 Hz. We used the reference respiration signal to evaluate EDR and measure vital signs and RSA indices, and we employed EDR for the tachogram decomposition algorithms. Informed consent was obtained from all subjects, and the institutional review board of the Korea Institute of Science and Technology (KIST) approved all procedures in this study (KIST-2019-015, July. 19, 2019). Informed consent was obtained from all participants prior to the experiment, and all experiments were conducted in strict accordance with KIST ethics guidelines and the declaration of Helsinki.

#### WESAD data

We used open-source data (WESAD, Wearable Stress and Affect Detection) to verify the proposed method in a public domain. This dataset contains the ECGs of 15 subjects (age = 27.5 ± 2.4 years), and its sampling rate is 700 Hz. The experimental protocol consisted of four different affective states (neutral, stress, amusement, meditation), and each emotion was mapped onto a two-dimensional affective space (arousal and valence) by using self-reports (SAM, Self-Assessment Manikins). The WESAD data are described in detail in^62^.

### Statistical evaluation

Since the simulation was performed with white Gaussian noise, ANOVA and t tests were used to compare the tachogram decomposition algorithms. In the case of real data (mindfulness meditation and WESAD data), nonparametric statistical tests were utilized because the number of subjects was not large. Specifically, the Wilcoxon signed rank test was employed when the two groups had the same sample size, and the Mann–Whitney U test was used otherwise. We defined p < 0.000005 as statistically significant. To depict the distribution, we utilized a box plot where the top and bottom boxes represent the 75th and 25th percentiles, and the center, top, and bottom lines represent the 50th, 90th, and 10th percentiles, respectively.

## Results

### Simulation

**Figure 4:**
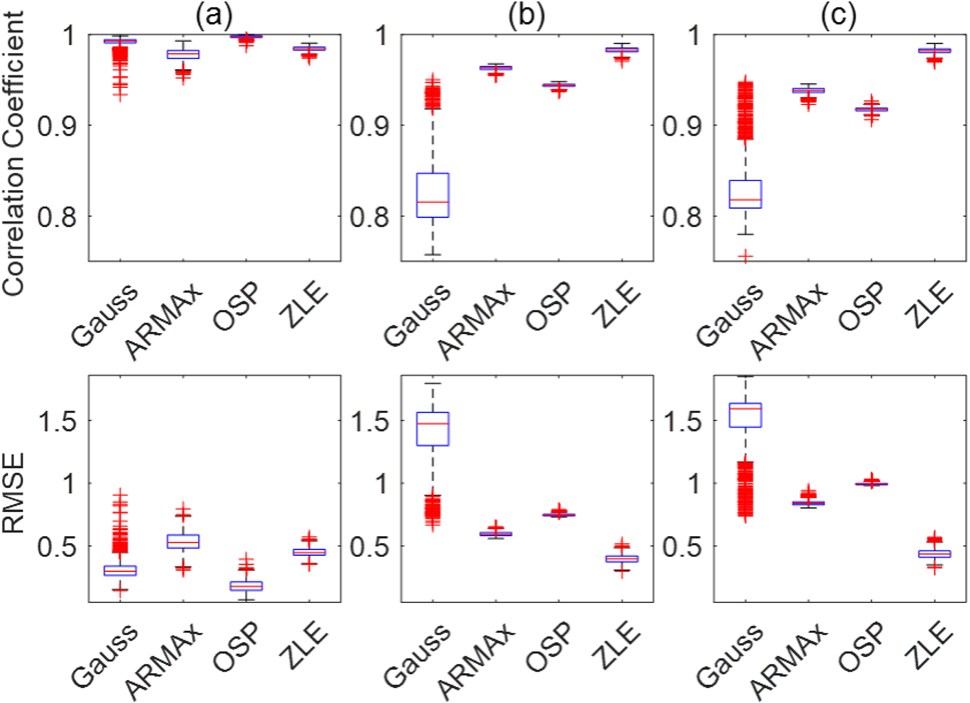
Correlation coefficient and RMSE between simulated ${\widetilde{{\varvec{x}}}}_{R-f}$ and filtered ${\overline{{\varvec{x}}} }_{R-f}$ (simulated RR: (**a**) constant, (**b**) linear function, and (**c**) sinusoidal function).

For each simulation type (RR: constant, linear function, and sinusoidal function), we calculated the Pearson correlation coefficient and root mean square error (RMSE) between $\widetilde{{\varvec{x}}}$_*R-f*_ and $\overline{{\varvec{x}} }$_*R-f*_ of the tachogram decomposition algorithms (Gauss, ARMAx, OSP, and ZLE). Figure 4 depicts the box plots of correlation coefficients and RMSEs ((a): a sine wave, (b): linear chirp, and (c): sinusoidal frequency modulated signal). For each simulation type, we conducted one-way ANOVA with Bonferroni correction to compare four algorithms (Gauss, ARMAx, OSP, and ZLE), and significant differences (p < 0.000005) were observed in both the Pearson correlation coefficients and RMSEs for all simulation types. For pairwise comparisons, post hoc tests were performed by using paired t tests, and all algorithm comparisons showed significant differences (p < 0.000005). The OSP showed the lowest RMSE and highest correlation coefficient when the simulated RR was constant (see Fig. 4a), but the ZLE had the lowest RMSE and highest correlation coefficient for other RR types (see Fig. 4b,c). It is notable that ZLE was robust to the fluctuation of instantaneous RR but contrastively Gauss did not work well in time-varying RR.

### EDR evaluation

We evaluated the performance of the aforementioned EDRs (EDR_A_, EDR_QR_, EDR_RS_, and EDR_SR_) by using the reference respiration signal of the mindfulness meditation data. Because there was a phase difference between EDR and reference respiration signal, we utilized two measures that were not significantly affected by the phase difference. One is the PLV between each EDR and the reference respiration signal, and the other is the mean absolute error (MAE) between RRs calculated by each EDR and the reference respiration signal. The PLV and MAE reflect the similarity of phases and the accuracy of RR estimation, respectively. As shown in Table 1, EDR_SR_ showed the best performance as in previous study^51^, so we employed EDR_SR_ in the following analysis.

**Table 1:** EDR evaluation.

Meditation phase	EDR_A_	EDR_QR_	EDR_RS_	EDR_SR_
PLV	0.7136	0.7389	0.7323	0.7410
MAE (Hz)	1.2927	0.8934	0.9700	0.8689

PLV and MAE between the reference respiration signal and each EDR (EDR_A_, EDR_QR_, EDR_RS_, and EDR_SR_). PLV and MAE indicate the phase synchronization and RR estimation error, respectively.

### Mindfulness meditation data

**Figure 5:**
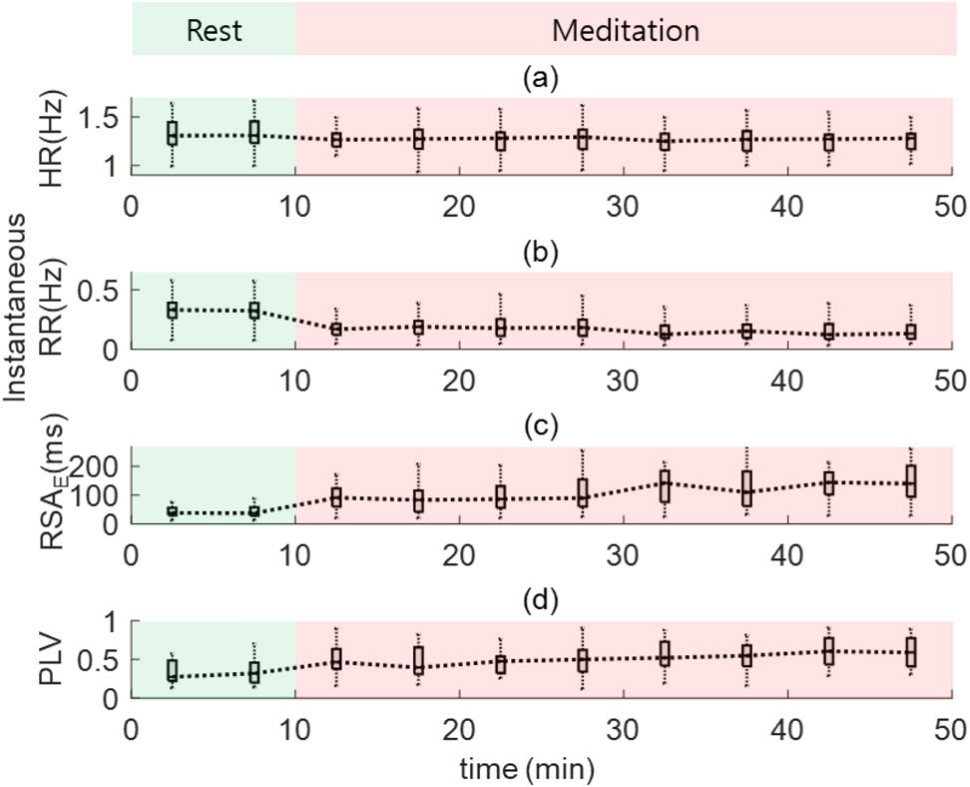
Vital signs (HR and RR) and RSA indices (RSA_E_ and PLV) for mindfulness meditation. Box plots of (**a**) HR, (**b**) RR, (**c**) RSA_E_, and (**d**) PLV.

**Figure 6:**
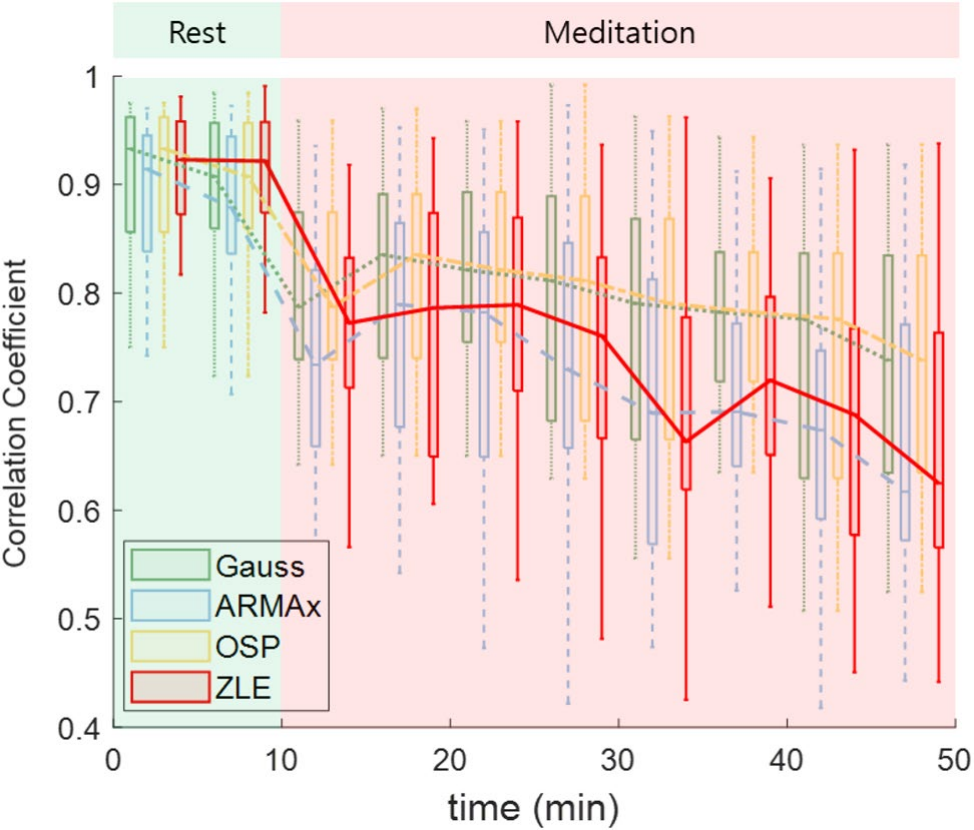
Correlation coefficient between raw tachogram (***x***_*RAW*_) and each RSA-free tachogram ($\overline{{\varvec{x}} }$_*R-f*_); $\overline{{\varvec{x}} }$_*R-f*_ of Gauss (green boxes and dotted line); $\overline{{\varvec{x}} }$_*R-f*_ of ARMAx (blue boxes and dashed line); $\overline{{\varvec{x}} }$_*R-f*_ of OSP (yellow boxes and dash-dotted line); $\overline{{\varvec{x}} }$_*R-f*_ of ZLE (red boxes and solid line).

Before applying the tachogram decomposition algorithms, we observed the effect of mindfulness meditation through various measures: VAS for stress, RSA indices, and vital signs. First, we confirmed the stress reduction after meditation through the VAS for stress (5.35 ± 1.77 → 2.47 ± 1.01). Second, we calculated the vital signs and RSA indices with 5 min intervals, and their box plots are depicted in Fig. 5. The horizontal lines represent the median values of the (a) HR, (b) RR, (c) RSA_E_, and (d) PLV. As meditation progressed, vital signs (HR and RR) decreased, and both the RSA_E_ and PLV increased. To assess the changes in the HR, RR, RSA_E_ and PLV, we performed statistical tests for these indices between the rest and meditation phases with the Mann–Whitney U test. Statistically significant differences (p < 0.000005) were observed in the instantaneous RR and RSA_E_. For each tachogram decomposition algorithm (ARMAx, OSP, and ZLE), we calculated Pearson correlation coefficients between ***x***_*RAW*_ and $\overline{{\varvec{x}} }$_*R-f*_, and their box plots and median values are shown in Fig. 6 (green boxes and dotted line: Gauss, blue boxes and dashed line: ARMAx, yellow boxes and dash dotted line: OSP, and red boxes and solid line: ZLE). A higher correlation coefficient indicates that $\overline{{\varvec{x}} }$_*R-f*_ is less filtered or less distorted.

**Figure 7:**
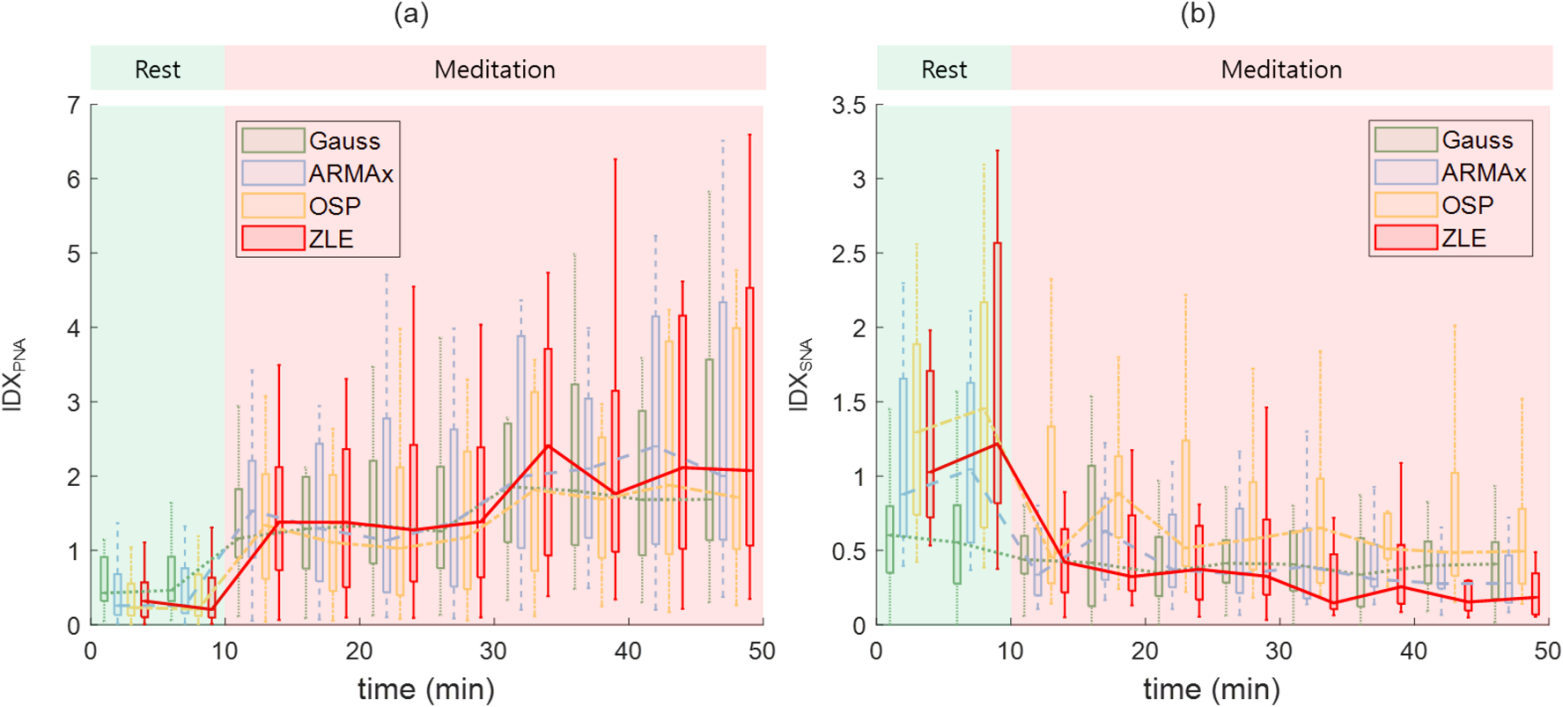
Changes in proposed HRV indices ((**a**): IDX_PNA_ and (**b**): IDX_SNA_) during mindfulness meditation. Gauss (green boxes and dotted line); ARMAx (blue boxes and dashed line); OSP (yellow boxes and dash-dotted line); ZLE (red boxes and solid line).

We computed the power spectra of tachograms and calculated HRV indices. Figure 7 shows the box plots and median values of the proposed HRV indices: (a) IDX_PNA_ and (b) IDX_SNA_. The boxes and lines represent (a) IDX_PNA_s and (b) IDX_SNA_s of Gauss (green boxes and dotted line), ARMAx (blue boxes and dashed line), OSP (yellow boxes and dash-dotted line), and ZLE (red boxes and solid line). To assess the trend of the HRV indices, we divided each record (50 min) into 1 rest phase and 4 meditation phases of equal length (10 min) and then compared the rest and each meditation phase by using a Wilcoxon’s two-sample signed rank test (see Table 2). Table 2 depicts the mean and standard deviation of HRV indices. HF and IDX_PNA_ are the HRV indices for vagal tone, and other indices (LF/HF, SB_U_ and IDX_SNA_) indicate sympathetic tone. No statistically significant differences were observed in HFs, but all IDX_PNA_s of ZLE were significantly increased during meditation (p < 0.000005). Unlike ZLE, IDX_PNA_s of previous tachogram decomposition algorithms did not significantly decrease at 20–30 min. For the HRV indices related to sympathetic tone, the LF/HF of the raw tachogram (***x***_*RAW*_) significantly increased at 30–50 min, which seems to be caused by RSA, but all SB_U_s and IDX_SNA_s of ZLE were significantly diminished (see Fig. 7; Table 2). Although, after 40 min, all SB_U_s and IDX_SNA_s of all algorithms except for Gauss were significantly decreased, but no statistically significant differences were observed in most SB_U_s and IDX_SNA_s of previous tachogram decomposition algorithms (Gauss, ARMAx, and OSP).

**Table 2:** Mean ± standard deviation of HRV indices (HF, IDX_PNA_, LF/HF, SB_U_, and IDX_SNA_).

HRV index	Tachogram decomposition algorithm	Rest (baseline)	10–20 min	20–30 min	30–40 min	40–50 min
HF	–	0.325 ± 0.356	0.453 ± 0.518	0.427 ± 0.465	0.456 ± 0.239	0.350 ± 0.351
IDX_PNA_	Gauss	0.572 ± 0.386	1.590 ± 1.266	1.741 ± 1.674	**2.642 ± 2.795**	**2.479 ± 2.172**
	ARMAx	0.445 ± 0.400	**1.718 ± 1.389**	1.921 ± 1.960	**2.825 ± 2.890**	**3.033 ± 2.718**
	OSP	0.396 ± 0.376	**1.498 ± 1.299**	1.668 ± 1.797	**2.368 ± 2.616**	**2.593 ± 2.540**
	ZLE	0.392 ± 0.354	**1.760 ± 1.567**	**1.866 ± 1.842**	**2.924 ± 3.229**	**3.056 ± 2.771**
LF/HF	–	2.793 ± 2.231	8.570 ± 12.101	9.234 ± 10.387	**13.545 ± 10.691**	**17.384 ± 17.838**
SB_U_	Gauss	1.850 ± 2.219	1.039 ± 0.994	0.864 ± 0.856	0.549 ± 0.450	0.629 ± 0.638
	ARMAx	1.629 ± 1.315	**0.675 ± 0.528**	0.952 ± 1.736	0.609 ± 0.640	**0.490 ± 0.490**
	OSP	3.067 ± 5.087	1.230 ± 1.092	2.142 ± 5.996	1.476 ± 2.142	**0.899 ± 0.866**
	ZLE	4.016 ± 9.224	**0.614 ± 0.481**	**0.626 ± 0.650**	**0.498 ± 0.704**	**0.402 ± 0.610**
IDX_SNA_	Gauss	0.611 ± 0.431	0.526 ± 0.409	0.454 ± 0.312	0.394 ± 0.276	0.433 ± 0.289
	ARMAx	1.155 ± 0.778	0.559 ± 0.407	0.779 ± 1.375	0.515 ± 0.486	**0.421 ± 0.369**
	OSP	1.561 ± 1.233	0.874 ± 0.637	1.464 ± 3.729	1.027 ± 1.278	**0.706 ± 0.618**
	ZLE	1.745 ± 1.732	**0.489 ± 0.332**	**0.497 ± 0.486**	**0.406 ± 0.506**	**0.336 ± 0.470**

Bold text represents a statistically significant difference between rest and each meditation phase (p < 0.000005). HF and IDX_PNA_ are the HRV indices for vagal tone, and other indices (LF/HF, SB_U_ and IDX_SNA_) indicate sympathetic tone. In all meditation phases, all IDX_PNA_s of ZLE were significantly increased, and statistically significant decreases were observed in all SB_U_s and IDX_SNA_s of ZLE.

### WESAD data

**Figure 8:**
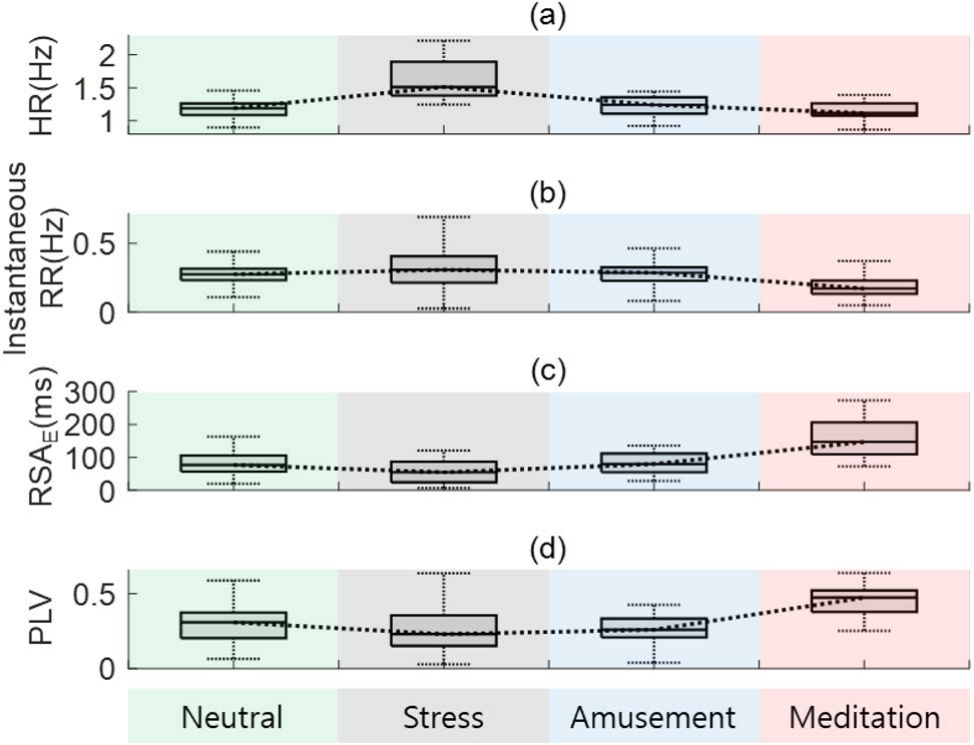
Vital signs (HR and RR) and RSA indices (RSA_E_ and PLV) for 4 affective states. Box plots of (**a**) HR, (**b**) instantaneous RR, (**c**) RSA_E_, and (**d**) PLV.

**Figure 9:**
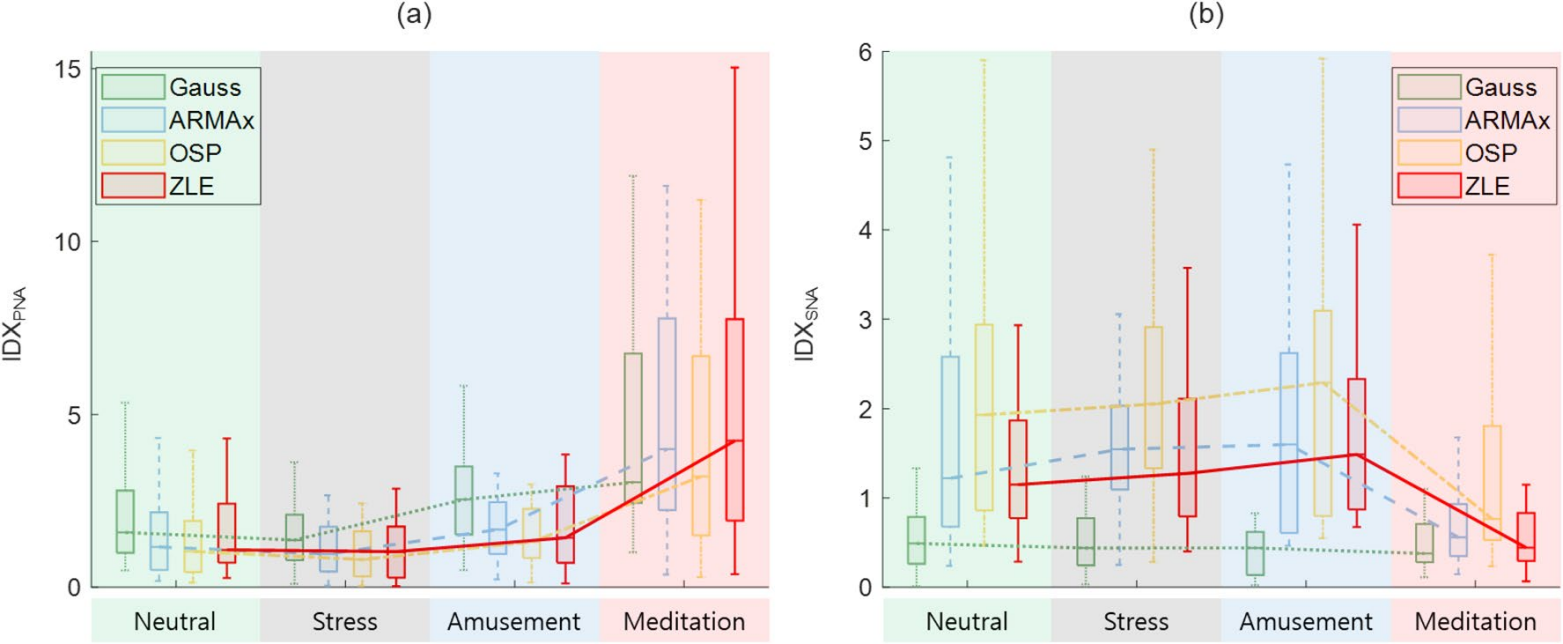
Changes in HRV indices ((**a**): IDX_PNA_ and (**b**): IDX_SNA_) of tachogram decomposition algorithms according to 4 affective states. Gauss (green boxes and dotted line); ARMAx (blue boxes and dashed line); OSP (yellow boxes and dash-dotted line); ZLE (red boxes and solid line).

The WESAD data contains ECGs with 4 affective states: neutral, stress, amusement, and meditation. The SAM self-reports showed that the lowest arousal scores (2.3 ± 1.4) occurred in the meditation state. As shown in Fig. 8, the RSA indices (RSA_E_ and PLV) and vital signs (HR and RR) had the highest and lowest values in the meditation state, respectively. For HR, RR, RSA_E_ and PLV, we performed statistical tests between the neutral and meditation states by using Mann–Whitney U tests. Statistically significant differences (p < 0.000005) were observed in all comparisons except HR. This is similar to the results of the previous subsection. Figure 9 depicts the box plots and median values of the proposed HRV indices: (a) IDX_PNA_ and (b) IDX_SNA_. The boxes and lines depict (a) IDX_PNA_s and (b) IDX_SNA_s of Gauss (green boxes and dotted line), ARMAx (blue boxes and dashed line), OSP (yellow boxes and dash-dotted line), and ZLE (red boxes and solid line). We evaluated the change between the neutral and other emotions of the HRV indices by using the Mann–Whitney U test. The mean and standard deviation of HRV indices are shown in Table 3. No statistically significant differences were observed in HFs, but IDX_PNA_ of ARMAx and ZLE significantly increased during meditation, in parallel with the RSA indices. Similar to the results of the previous subsection, SB_U_ and IDX_SNA_ of the ZLE significantly (p < 0.000005) decreased during meditation, but no statistically significant differences were observed in SB_U_ and IDX_SNA_ of other decomposition algorithms (Gauss, ARMAx and OSP) as shown in Fig. 9 and Table 3.

**Table 3:** Mean ± standard deviation of HRV indices (HF, IDX_PNA_, LF/HF, SB_U_, and IDX_SNA_).

HRV index	Tachogram decomposition algorithm	Neutral (baseline)	Stress	Amusement	Meditation
HF	–	1.376 ± 1.546	1.062 ± 1.691	1.325 ± 1.425	2.287 ± 2.131
IDX_PNA_	Gauss	2.306 ± 1.971	1.781 ± 1.699	2.972 ± 2.062	5.075 ± 4.252
	ARMAx	1.672 ± 1.623	1.394 ± 1.712	1.930 ± 1.619	**5.834 ± 5.562**
	OSP	1.521 ± 1.527	1.242 ± 1.600	1.679 ± 1.483	5.051 ± 5.222
	ZLE	1.731 ± 1.609	1.395 ± 1.551	2.049 ± 1.975	**5.776 ± 5.131**
LF/HF	–	2.897 ± 2.142	3.909 ± 3.856	5.173 ± 4.474	4.971 ± 5.749
SB_U_	Gauss	1.275 ± 1.168	1.385 ± 1.686	1.273 ± 2.146	0.943 ± 0.741
	ARMAx	5.746 ± 9.775	4.194 ± 3.190	6.431 ± 9.649	1.235 ± 1.225
	OSP	10.621 ± 19.805	9.131 ± 9.516	15.591 ± 26.228	2.684 ± 3.565
	ZLE	2.640 ± 1.671	37.599 ± 112.521	4.267 ± 4.604	**0.809 ± 0.660**
IDX_SNA_	Gauss	0.533 ± 0.388	0.631 ± 0.577	0.523 ± 0.625	0.539 ± 0.476
	ARMAx	1.897 ± 1.624	1.725 ± 1.051	1.820 ± 1.431	0.830 ± 0.722
	OSP	2.235 ± 1.813	2.184 ± 1.237	2.473 ± 2.169	1.477 ± 1.829
	ZLE	1.477 ± 0.969	1.872 ± 1.595	2.195 ± 2.393	**0.610 ± 0.490**

Bold text represents a statistically significant difference between neutral and other affective states (p < 0.000005). HF and IDX_PNA_ are the HRV indices related to vagal tone, and other indices (LF/HF, SB_U_ and IDX_SNA_) indicate sympathetic tone. In meditation state, IDX_PNA_s of ARMAx and ZLE showed significant increases, and statistically significant decreases were observed in SB_U_s and IDX_SNA_s of ZLE.

## Discussion

The tachogram of HRV consists of various oscillations caused by complicated interactions between the heart and brain. In particular, the oscillation caused by RSA often forms most of the raw tachogram ***x***_*RAW*_. For the precise quantification of autonomic nervous system activity, it is necessary to decompose the raw tachogram into RSA and RSA-free tachograms. In this study, we proposed the novel tachogram decomposition algorithm (ZLE) and new HRV indices (IDX_PNA_ and IDX_SNA_). We evaluated the performance of the proposed method through simulations, mindfulness meditation data, and WESAD data.

RSA, which is a synchrony between the heartbeat interval and breathing, reflects cardiac vagal control. To quantify RSA, we employed the PLV and RSA_E_. Although the RSA_E_ and PLV capture different features (phase interaction and RRI variation), these indices increased during meditation (see Figs. 6, 9) as in previous study^39^, which indicates parasympathetic activation. In HRV analysis, all IDX_PNA_s of ZLE showed significant increases in contrast to HFs (see Tables 2, 3), and IDX_PNA_ appeared to be better than HF in identifying parasympathetic activation.

Sympathetic inhibition during meditation is supported not only by literature but also by experimental analysis. Breathing meditation and slow breathing have been reported to improve pulmonary gas exchange performance^63^ and lower blood pressure^64^, galvanic skin response^65^ and muscle sympathetic nerve activity^66–68^. Reductions in vital signs (HR and RR) and self-report scores (stress and arousal) were also observed during meditation in our experiments, as shown in Figs. 6 and 9. Despite much evidence for sympathetic inhibition, the LF/HF of the classical HRV was noticeably increased during meditation^35–40^ (see Tables 2, 3); it seems to be due to the influence of RSA. To cancel the interference of RSA, C. Varon et al. suggested SB_U_^46^ and we proposed IDX_SNA_. Although SB_U_ (LF_*R-f*_/(LF_*RSA*_ + HF_*RSA*_)) is similar to IDX_SNA_ (LF_*R-f*_/(LF_*RSA*_ + HF_*RSA*_ + HF_*R-f*_)), IDX_SNA_ includes RSA-free vagal tone information (HF_*R-f*_). Since RSA is insufficient to represent a whole cardiac vagal tone^69^, the proposed IDX_SNA_ can be considered a more reasonable index to quantify sympathetic tone. In real meditation data, both SB_U_ and IDX_SNA_ of ZLE successfully identified sympathetic inhibition, and it may be the first report on the identification of cardiac sympathetic inhibition during meditation. As a result, both SB_U_ and IDX_SNA_ appear to be more reliable than classical LF/HF to identify sympathetic inhibition.

ARMAx and OSP share the same scheme, which finds a weight vector (w = (*RESP*^*T*^*∙RESP*)^−1^*RESP∙X*) and subtracts $\overline{{\varvec{x}} }$_*RSA*_ from the original tachogram ***x***_*RAW*_^45^. When the *RESP* is the trajectory matrix of EDR, the ARMAx and OSP can be interpreted as adaptive noise cancellers with a least square solution; then, the EDR and the RSA component (***x***_*RSA*_) can be regarded as a noise reference signal and true noise, respectively. The algorithm performance is determined by a linear correlation between ***x***_*RSA*_ and the EDR^70^, but their true relationship is nonlinear^47^. Specifically, both the amplitude and frequency of the original tachogram are modulated by the RR and tidal volume^27–29^, and such modulations cannot be expressed by a linear transform of EDR. As an alternative, Gauss was suggested, which assumes paced respiration^48^, but instantaneous RR generally changes with time. In the simulation, previous decomposition algorithms (Gauss, ARMAx, and OSP) performed well when RR was constant, but their performance was insufficient in time-varying RR. However, ZLE was robust to the fluctuations of simulated RR (see Fig. 4). Note that abrupt changes in RR often occur during breathing meditation. In fact, the HRV indices (IDX_PNA_, SB_U_, and IDX_SNA_) of ZLE certainly identified both parasympathetic activation and sympathetic inhibition during meditation when no statistically significant differences were observed in some IDX_PNA_s and most IDX_SNA_ of both ARMAx and OSP (see Tables 2, 3). Therefore, the ZLE seems to be the most reliable tachogram decomposition algorithm when RR dynamically changes.

Despite the promising performance of the proposed ZLE and HRV indices, they have limitations. First, if some oscillation of the RSA-free tachogram (***x***_*R-f*_) overlaps with the stop band of the notch filter in the frequency domain, then the corresponding oscillation will be suppressed. This filtering loss can be observed through the correlation coefficient between ***x***_*RAW*_ and $\overline{{\varvec{x}} }$_*R-f*_ when the influence of RSA was small (e.g., resting state). As shown in Fig. 6, the loss of ZLE seems to be insignificant. Second, the proposed method did not identify sympathetic activation. In the stress state, sympathetic nerves are activated, but none of the SB_U_s or IDX_SNA_s show a significant increase. In future works, we plan to advance the proposed method for stress assessment.

## Conclusions

To precisely assess autonomic nervous system activity, we proposed a novel tachogram decomposition algorithm (ZLE) and new HRV indices (IDX_PNA_ and IDX_SNA_). ZLE clearly decomposed ***x***_*RAW*_ into $\overline{{\varvec{x}} }$_*RSA*_ and $\overline{{\varvec{x}} }$_*R-f*_, and IDX_PNA_ and IDX_SNA_ identified parasympathetic activation and sympathetic inhibition during meditation, respectively. This study may be the first report on the identification of cardiac sympathetic inhibition during meditation (or slow breathing). Although the overlapping issue of the ZLE still remains, the ZLE was more robust than previous tachogram decomposition algorithms (Gauss, ARMAx and OSP) when RR dynamically fluctuates. Since the proposed approach requires only a single-lead ECG, we expect that it will be used in various fields, such as the internet of Medical Things (IoMT), digital healthcare, digital meditation, and digital therapeutics.

## Data Availability

The datasets used and/or analysed during the current study available from the corresponding author on reasonable request.
